# Transcriptional and post-transcriptional events trigger *de novo infB* expression in cold stressed *Escherichia coli*

**DOI:** 10.1093/nar/gkz187

**Published:** 2019-03-27

**Authors:** Anna Brandi, Mara Giangrossi, Silvia Paoloni, Roberto Spurio, Anna M Giuliodori, Cynthia L Pon, Claudio O Gualerzi

**Affiliations:** Laboratory of Genetics, Department of Biosciences and Biotechnology University of Camerino, 62032 Camerino (MC), Italy

## Abstract

After a 37 to 10°C temperature downshift the level of translation initiation factor IF2, like that of IF1 and IF3, increases at least 3-fold with respect to the ribosomes. To clarify the mechanisms and conditions leading to cold-stress induction of *infB* expression, the consequences of this temperature shift on *infB* (IF2) transcription, *infB* mRNA stability and translation were analysed. The *Escherichia coli* gene encoding IF2 is part of the *metY-nusA-infB* operon that contains three known promoters (P-1, P0 and P2) in addition to two promoters P3 and P4 identified in this study, the latter committed to the synthesis of a monocistronic mRNA encoding exclusively IF2. The results obtained indicate that the increased level of IF2 following cold stress depends on three mechanisms: (i) activation of all the promoters of the operon, P-1 being the most cold-responsive, as a likely consequence of the reduction of the ppGpp level that follows cold stress; (ii) a large increase in *infB* mRNA half-life and (iii) the cold-shock induced translational bias that ensures efficient translation of *infB* mRNA by the translational apparatus of cold shocked cells. A comparison of the mechanisms responsible for the cold shock induction of the three initiation factors is also presented.

## INTRODUCTION

During the acclimation period that follows a cold-shock (i.e. an abrupt temperature downshift from 37° to <20°C), the growth of *Escherichia coli* cells is temporarily interrupted, the fluidity of the cellular membrane decreases, DNA supercoiling and compaction increase, bulk transcription and translation are blocked and a small set of genes coding for ‘cold shock’ proteins is preferentially expressed (1–5).

A cold stress-induced modification of the translational apparatus defined ‘cold-stress translational bias’ is at the root of the preferential translation of the cold-shock transcripts. This bias largely depends on an increased level of translation initiation factors IF3 and IF1 with respect to ribosomes (5–12). The third factor (IF2) does not seem to be involved in the translational bias but also its level increases after the stress (12). As a result of these increases and of the concomitant drastic reduction of the synthesis and assembly rate of the ribosomal components during the cold acclimation period that follows the stress, the IFs/ribosomes stoichiometric ratio, which is otherwise kept constant at 0.12–0.15:1 under normal growth conditions (13), undergoes a substantial imbalance becoming as high as 0.45–0.50:1 (12).

The transcriptional and post-transcriptional mechanisms leading to the cold-stress induction of *infA* (IF1) (9) and *infC* (IF3) (10) expression have been at least in part clarified. In fact, it has been shown that only one of the promoters directing *infA* transcription and one of *infC* are preferentially activated by cold stress and that the resulting mRNAs have longer half-lives (*t*_1/2_) and are preferentially translated by the translational machinery of cold-shocked cells whereas translational auto-repression by IF3 is reduced (9,10,12). By contrast, nothing is known concerning the mechanism that determines the cold-shock induction of *infB* (IF2).

**Figure 1.**
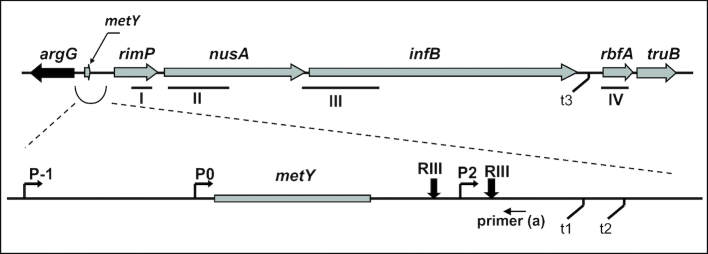
Scheme of the *Escherichia coli* chromosomal region corresponding to the *nusA-infB* operon. The region surrounding *metY* is enlarged to show better the location of the promoters identified so far. The positions of P-1, P0 and P2 promoters, the transcription terminators (t1, t2 and t3) as well as the RNaseIII processing sites are indicated in the scheme. The black arrow indicates the region corresponding to the oligonucleotide primer (a) used in primer extension analyses. The bars designated with roman numbers indicate the sequences corresponding to the probes used for northern blot analyses. Further details are given in the text.

As schematically sketched in Figure 1, *E. coli infB* is located in the *nusA-infB* operon (14–17) at 68.9 min in the genetic linkage map (18) adjacent to the *rpsO* and *pnp* operons (17,19). This chromosomal region contains several other genes (*nusA, infB, rbfA, rpsO, pnp*) whose expression is induced by cold stress (1,5). In *E. coli K12*, the polycistronic *metY-nusA-infB* transcriptional unit encodes tRNA_2_^fMet^, a minor form of the initiator tRNA (15,20,21) not essential for cellular growth (21) and not present in some *E. coli* strains (see below). Upon translation, this polycistronic RNA yields: (i) RimP (formerly YhbC or P15a) a protein involved in the maturation of the 30S subunit (22) by allowing a faster binding of proteins S9 and S19 while inhibiting the binding of S12 and S13 (23), (ii) the transcription termination factor NusA (24), which acts as an RNA chaperone coordinating 16S rRNA folding and RNase III processing during 30S production (25), (iii) the two forms (α and β) of IF2 (26) whose involvement in ribosome assembly/maturation is illustrated in the accompanying article (Brandi *et al.*), (iv) RbfA, another protein involved in the assembly of the 30S ribosomal subunit (27,28) and (v) TruB, the pseudouridine-55 (psi55) synthase responsible for modifying U55 of tRNA molecules (29). The clustering of these genes within the same operon is therefore consistent with the circumstance that all these gene products play a role in the same or in related functions dealing with ribosome biogenesis.

The primary transcripts of this operon consist of a pre-tRNA molecule physically linked, at least initially, to mRNA sequences encoding the aforementioned proteins. Furthermore, some transcripts of this operon extend into the downstream *rpsO* and *pnp* operons, which encode ribosomal protein S15 and polynucleotide phosphorylase (17,19,30).

Multiple promoters, transcription terminators and RNA processing sites have been detected within the *metY-nusA-infB* operon (Figure 1): P-1 and P0, located upstream of *metY* and P2, an internal promoter located at the 3′ side of *metY* (15,20,31).

In this study the consequences of a temperature shift (37°C →10°C) on *infB* transcription, mRNA stability and translation were analysed with the aim of clarifying mechanisms and conditions that lead to the cold-stress induction of *infB* expression. We report on the elucidation of the transcriptional or post-transcriptional regulations of *infB* expression, the detection of new promoters and the identification of the promoter(s) preferentially used during cold adaptation.

## MATERIALS AND METHODS

### Strains

*Escherichia coli* strains used in this work were: MRE600; DH5α; MC4100; MG1673 (LAM-, thyA715, rph-1); SK7622 (LAM-, thyA715, rph-1, rncΔ38::Kan) (32).

### Semi-quantitative analysis of IF2 levels by western blotting

*Escherichia coli* cells were disrupted by heating at 100°C in Laemmli sample Buffer. Total protein aliquots (20 μg each), as quantified (33), were subjected to 8% sodium dodecylsulphate-polyacrylamide gel electrophoresis (SDS-PAGE) followed by electroblotting in 50 mM Tris–HCl (pH 8.3), 80 mM glycine, 0.04% SDS and 20% methanol onto a cellulose nitrate membrane using the Sammy Dry System (Schleicher & Schuell) for 60 min at constant amperage (2.5 mA/cm^2^). IF2 was detected by rabbit polyclonal anti-IF2 antibodies and quantified essentially as described (6).

### Northern blotting

At the indicated times, aliquots of *E. coli* strains growing in Luria Bertani (LB) medium at 37°C or subjected to cold shock were withdrawn for total RNA extraction. After centrifugation the cell pellet was washed in NaCl 0.9%, resuspended in hot lysis buffer (100 mM Tris–HCl, pH 8.0; 2 mM ethylenediaminetetraacetic acid (EDTA); 1% SDS) and maintained for 3 min at 95°C with repeated vortexing. The cell lysates were then incubated for 10 min in ice, followed by addition of cold 2 M KCl (to a final concentration 900 mM). After an additional 5 min in ice, the lysates were clarified by centrifugation and the RNA extracted with hot phenol and chloroform:isoamyl alcohol (24:1). Total RNA was then precipitated with three volumes of ethanol in 1 M LiCl. Aliquots of 8 μg of total RNA were subjected to formaldehyde agarose gel electrophoresis, blotting and hybridization (9) with probes obtained by polymerase chain reaction (PCR) amplification of chromosomal DNA using the primer pairs indicated in Supplementary Table S1 and ^32^P-labelled by random primer reaction (34). The radioactivity of the DNA–mRNA hybrids was quantified by a Personal Molecular Imager FX (Bio-Rad) and normalized as a function of the specific radioactivity of the individual probes and to the amount of 16S rRNA present in each sample quantified by ethidium bromide (Supplementary Figure S1) and by quantification of the radioactivity resulting from hybridization with a complementary oligonucleotide probe.

### Assessment of the chemical stability of *infB* transcript

Cultures of *E. coli* MRE600 in exponential phase of growth at 37°C were treated with Rifampicin (250 μg/ml) before or after cold stress as specified in the legend to Figure 4. Aliquots from the cultures were harvested at the times indicated in the abscissae of the corresponding figures; total RNA was extracted and analysed for the presence of *infB* mRNA by northern blot and hybridization with a radioactively labelled probe III (Figure 1). The blotting and the hybridization reactions were as described (6, 9).

### Primer extension analyses

Primer ‘a’ is complementary to the DNA region downstream of RNaseIII sites indicated in Figure 1, and its sequence is 5′-TAACTGAACCCTATAACCGCAAC-3′. Primer ‘b’ corresponds to a region downstream the HindIII site of the pKK232–8 polylinker and its sequence is 5′-GCTCCTGAAAATCTCGT CGAAGCTCG-3′. The reactions were carried out on total RNA (8–12 μg) denatured at 65°C for 10 min and allowed to anneal at 48°C for 30 min with 3 pmol of 5′-end-[^32^P] labelled primers in a 10 μl reaction mixture containing 100 mM dNTPs and 3–4 units of AMV reverse transcriptase (Amersham). The reaction products were analysed on 7% PAGE-Urea (7 M) gel in TBE buffer run in parallel with a sequencing reaction performed by the Sanger dideoxy chain termination reaction (34) using the same oligonucleotide primer.

### Construction of *nusA-infB* transcriptional fusions

The transcriptional fusions were obtained by ligating to the promoterless *cat* gene in pKK232–8 (Pharmacia) DNA fragments of different lengths obtained by PCR of *E. coli* MRE600 DNA. The primer pairs and the annealing temperature used in each amplification reaction are listed in Supplementary Table S2. The pKKAB constructs were obtained by cloning the PCR products in the SmaI site of pKK232–8 polylinker followed by direct selection on chloramphenicol of cells transformed with the recombinant pKK232–8 containing a functional promoter sequence upstream of the*cat* gene. The construct pKK232MG1 was obtained cloning in BamHI and HindIII polylinker sites the 390 bp PCR product digested with the same enzymes.

The construct pKK232MG2 was obtained by digesting the 390 bp PCR product with EcoRV/HindIII to obtain a 130 bp DNA fragment that was cloned into the SmaI and HindIII polylinker site upstream of the promoterless *cat* gene. *Escherichia coli* DH5α competent cells were transformed with these recombinant plasmids and selected for chloramphenicol (CAM) resistance.

### Construction of pTZ18infB

A DNA fragment (∼2.9 kbp) containing the entire *infB* coding region plus short flanking sequences was excised from pPLC*infB* (35) by digestion with EcoRI/HindIII and ligated into the corresponding sites of pTZ18R (Pharmacia). The DNA fragment cloned into pTZ18 corresponds to the region comprised between 3313176 and 3316155 (reverse strand) of the genomic sequence NC_000913.3 of *Escherichia coli* str. K-12 substr. MG1655.

### *In vitro* translation

The *infB* mRNA used for the *in vitro* translation tests was prepared from pTZ18*infB* by *in vitro* transcription with T7 RNA polymerase (36). Cell extracts were prepared as described (36) from *E. coli* MRE600 grown in LB medium at 37°C to *A*_600_ = 1 (ncs S30 = non-cold-shock S30) and after 90 min of incubation at 10°C (cs S30 = cold-shock S30) and incubated with the amounts of *infB* mRNA indicated in the abscissa of Figure 8. The reaction mixtures (30 μl) were supplemented with [^35^S] methionine and with the other 19 non-radioactive amino acids, GTP, ATP and an ATP regenerating system as described (37). As explained below (legend to Figure 8), *infB* mRNA translation was also carried out in parallel in systems containing [^14^C] tyrosine or [^14^C] phenylalanine (Supplementary Figure S8) as precursors in place of [^35^S] methionine. At the end of 30-min incubation at 37 or 15°C, 5 μl aliquots were used for determination of the hot-trichloroacetic acid (TCA)-insoluble radioactivity incorporated whereas the remaining 25 μl were subjected to SDS-PAGE analysis. To identify the nature of the product synthesized, the resulting gel was subjected to autoradiography and to western blotting using a polyclonal anti-IF2 antibody.

### tRNA purification and electrophoresis

Total tRNA was isolated from 10 ml of an *E. coli* culture (38) and stored in TE buffer (10 mM Tris–HCl, pH 8, 1 mM EDTA, pH 8) at −80°C. Single aliquots of tRNA (∼50 μg) were mixed with 4 μl of sample buffer (final concentration 0.03 μg ethidium bromide, 5 mM EDTA pH 8, 5% glycerol, 0.005% xylene cyanol and 0.005% bromophenol blue) and separated by electrophoresis (39) on a 15% non-denaturing polyacrylamide gel (30:1 acrylamide:bisacrylamide) in TBE 1.8× (1× = 50 mM Tris, 50 mM Boric acid, 1 mM EDTA pH8) at 500 V (∼35 V/cm). After an overnight run at room temperature, the portion of the gel between the two dyes was electro-blotted onto Hybond-N membrane (Amersham) using a Hoefer Electroblot apparatus at 2 mA/cm^2^ for 150 min with 40 mM Tris-acetate, 2 mM Na_2_EDTA (pH 8.1). The membrane was hybridized at 40°C (34) with a 5′-end-[^32^P]-labelled oligonucleotide (5-′AAC CGA CGA TCT TCG GG-3′) specific for *metY/metZ* detection.

## RESULTS

### Steady state level of *infB* transcript before and after cold stress

**Figure 2.**
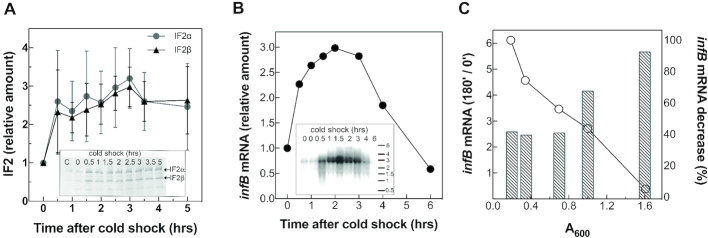
Variation of the cellular levels of IF2 and *infB* mRNA as a function of cell density and following cold stress. (**A**) Relative increase of IF2α (•) and IF2β (▴) levels at the indicated times after a cold stress induced by shifting from 37 to 10°C a cell culture that had attained *A*_600_ = 0.5 with respect to the pre-shock level (time 0) taken as = 1.The amount of IF2 was estimated semi-quantitatively by subjecting western blots developed using a rabbit polyclonal anti-IF2 serum as described (6) to densitometry using a BioRad imaging densitometer GS-670. The error bars refer to the standard deviation calculated on the relative increase of the IF2 level from results obtained in three separate experiments carried out in duplicate. The inset shows a typical western blot of purified IF2α and IF2β (lane C) and of the factor present in extracts of cells at the onset of cold stress (time 0) and at the times of cold stress indicated above each lane. (**B**) Relative increase of the *infB* mRNA level at the times after the cold shock indicated in the abscissa with respect to the pre-shock (time 0) level taken as = 1. The amount of the mRNA was quantified from the radioactivity of the northern blot bands as detected by a BioRad FX molecular imager. A typical northern blot is presented in the inset; the numbers above the individual lanes indicate the time (hours) elapsed since the onset of the cold stress that is indicated as ‘0’. An RNA ladder (Thermo Scientific RoboRuler High Range RNA Ladder 200–6000 bases) is presented on the right side; (**C**) decreasing level of *infB* mRNA (○) as a function of the cell density (reported in the abscissa) of a culture growing in LB at 37°C. The decreasing *infB* mRNA level is expressed on the right side *y*-axis as %, taking as 100% the level detected at cell density *A*_600_ = 0.2. The histogram bars (left *y*-axis) indicate the ratios between the *infB* mRNA levels in cells cold-stressed for 180 min at the cell density indicated in the abscissa and the levels determined before cold-shock (time 0) induced at the corresponding cell density.

As mentioned above, compared to the pre-stress level, the amount of IF2 increases considerably in cells during the cold acclimation phase that follows an abrupt temperature downshift from 37 to 15 or 10°C (Figure 2A).

The extent of the IF2 increase (∼3-fold) occurring after cold stress is in good agreement with published results (7) of a pulse-labelling experiment that demonstrated that the incorporation of radioactive methionine in the IF2 molecule increases 3–3.5 times between 1.5 and 3 h after a 37 to 10°C temperature downshift.

As expected, the increase of the IF2 level occurring after cold stress is paralleled by an increase of the level of the *infB* mRNA that encodes the factor, as seen in Figure 2B. Several northern blots similar to that shown in Figure 2B and yielding similar results were obtained with the same *E. coli* strain (MRE600). The results of two of these experiments are shown in Supplementary Figure S2. *Escherichia coli* MRE600, a derivative of *E. coli* B, had always been used in our previous translational studies and for the sake of homogeneity the same strain was used also in the present study. Nevertheless, to ascertain that the results obtained with MRE600 are of general relevance and not bacterial strain specific, parallel experiments were also performed using two K12 derivative strains (i.e. MG1673 and SK7622). The results obtained with these strains show that the steady state level of *infB* mRNA increases during cold acclimation approximately to the same extent observed with MRE600 (Supplementary Figure S3).

Because the extent of the *infB* mRNA and IF2 increases are similar, it can be argued that translation initiation as well as ribosome recycling on *infB* mRNA are not very efficient during cold adaptation. Finally, it should be noted that the extent of the *infB* mRNA increase depends upon the basal level of this RNA at the time of cold-shock, which in turn depends upon the cell density of the culture (Figure 2C).

### Analysis of the operon transcripts

The proximal portion of the *nusA-infB* operon contains three promoters, two of them (P-1 and P0) upstream and the other (P2) downstream of *metY* (Figure 1). Transcription from any one of these promoters would produce a polycistronic RNA encoding *rimP, nusA, infB* and *rbfA* and could be responsible for the increased level of the *infB* transcript. However, the transcriptional pattern is made much more complex by the presence of at least three transcription terminators, two (t1 and t2) upstream of *rimP* and another (t3) upstream of *rbfA* (40), by the possible existence of other so far unidentified promoters (see below) and by two RNaseIII processing sites (20,40,41). Furthermore, the operon encodes the factor NusA that controls transcription termination/anti-termination events and plays a role regulating its own expression as well as that of the entire operon, including *infB*. Indeed, it had been shown that the level of all these transcripts increases in mutants in which transcription termination by NusA is reduced (42).

In light of the above considerations, the nature of the transcripts produced by the *nusA-infB* operon was investigated by subjecting the total RNA extracted from cells before and after cold stress to northern blot analyses using probes specific for *rimP, nusA, infB* and *rbfA* as well as *metY* (Figure 1).

**Figure 3.**
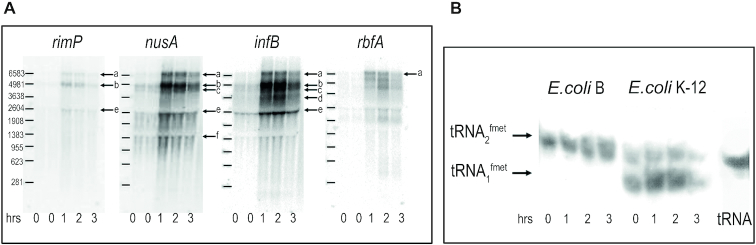
Analysis of the RNA transcripts of the *nusA-infB* operon before and after cold stress. (**A**) Analysis of the transcripts recognized by the probes indicated in Figure 1 hybridizing specifically with: from left to right *rimP* (probe I), *nusA* (probe II), *infB* (probe III) and *rbfA* (probe IV) genes. The arrows labelled with letters ‘a’ though ‘f’ indicate specific RNA molecules whose nature is discussed in the text. (**B**) Electrophoretic analysis of the tRNA_i_ transcribed before and after cold stress from *metY* and *metZ* present in *Escherichia coli* K-12 and MRE600, respectively. The time elapsed after a 37 to 10°C cold stress is indicated below each lane. Further details are given in the text.

Several discrete bands, corresponding to RNA molecules of different sizes, were detected by radioactive probes specific for the aforementioned protein-encoding genes (Figure 3A). As judged from the intensity of the bands hybridized to the individual probes, and after taking into account the differences in the specific radioactivity of the probes used for hybridization and normalization for the amount of 16S rRNA, it can be estimated that before cold stress the mRNA levels corresponding to individual genes of the operon were *nusA* > *infB* > *rimP* > *rbfA*. This finding is in good agreement with a previous study in which the number of *infB* mRNA molecules/cell was reported to be slightly higher compared to *rimP* mRNA, about twice that of *rbfA* mRNA and less than half that of *nusA* mRNA (43). After cold stress, the intensity of all transcript bands is strongly increased, indicating that transcription of the entire operon is activated by the stress; furthermore, the northern blot patterns are slightly different after the stress, suggesting that the activity of the various promoters and/or terminators becomes somewhat modified (Figure 3A).

The largest (∼6.7 kb) of the electrophoretically resolved RNA molecules (band ‘a’) is recognized by the probes specific for all four protein-encoding genes and therefore corresponds to a transcript originating from one or more of the promoters (P-1, P0 and P2) present in the proximal portion of the operon and extending as far as *rbfA* so as to include the *rimP, nusA, infB* and *rbfA* sequences (Figure 1). The second largest (∼4.8 kb) RNA (band ‘b’) is not recognized by the *rbfA*-specific probe and likely represents a transcript containing only *rimP, nusA* and *infB* and terminating at the t3 site just upstream of *rbfA* (Figure 1). A somewhat smaller RNA molecule (band ‘c’) migrating just below band ‘b’ is recognized by both *nusA-* and *infB-*specific probes but not by the probes directed against *rimP* and *rbfA*. The hybridization property and the size of this molecule (∼4.3 kb), which corresponds well to the combined size of NusA and IF2, indicate that this mRNA encodes exclusively these two proteins. It is remarkable that there is only a small amount of this transcript in the samples taken before cold stress. This observation suggests that upstream of *nusA* there might be a cold-inducible promoter never detected before (see below). The fourth largest (∼3.6 kb) transcript (band ‘d’) and the band ‘e’ (∼2.7 kb) are recognized by the *infB*-specific probe and their size is compatible with that expected for mRNAs encoding IF2. This finding suggests that *infB* might be transcribed not only as a polycistronic but also as a monocistronic mRNA from a promoter located just upstream of this gene and never detected before (see below). Finally, an additional RNA molecule is clearly detectable in the northern blot. This band ‘f’ (∼1.4 kb) hybridizes only with the *nusA* probe and its size roughly corresponds to that expected for a transcript of the *nusA* gene (1.113 kb).

In addition to encoding the aforementioned proteins, the *nusA-infB* operon contains a gene encoding initiator tRNA*_i_* (tRNA^fmet^). Indeed, in *E. coli* K-12 about 25% of the total tRNA*_i_* is the product of *metY* located at the beginning of the operon. This gene encodes a minor form of initiator tRNA that bears an A instead of m^7^G at position 46, whereas the remaining 75% derives from three identical, tandemly organized genes (21,44) located at 63.5 min (18). However, in *E. coli B* strains, such as *E. coli* MRE600 used in some of the present experiments, *metY* is replaced by a gene encoding an initiator tRNA identical to the other three (21,44,45). The expression of *metY* before and after cold stress was investigated by northern blotting performed after electrophoretic separation of the two types of tRNA_i_ (i.e. tRNA_1_^fmet^ and tRNA_2_^fmet^) (39). As seen in Figure 3B, in *E. coli* K-12 after 2–3 h of cold stress the product of *metY* is considerably increased compared to the pre-stress level of tRNA_2_^fmet^. Because tRNA molecules are known to be fairly stable, it seems unlikely that the increased tRNA level results from an increased half-life; this finding indicates instead that either one or both *metY* upstream promoters (P-1 and P0) are activated by the cold stress. However, a different result was obtained when the same experiment was carried out on RNA extracted from *E. coli* MRE600 in which *metY* is replaced by *metZ* as described above. In this case, the total level of tRNA_i_ represented exclusively by tRNA_1_^fmet^ remains more or less constant after cold stress (Figure 3B). A possible explanation for this finding is given in ‘Discussion’ section.

### Determinants of the increased IF2 expression during cold acclimation

As in the case of other cold-shock proteins (6–9), the increased level of IF2 in cells could be due to several not mutually exclusive reasons. Indeed, cold-shock may induce *infB* transcription, may increase the mRNA stability or may increase the translational efficiency of the *infB* mRNA.

**Figure 4.**
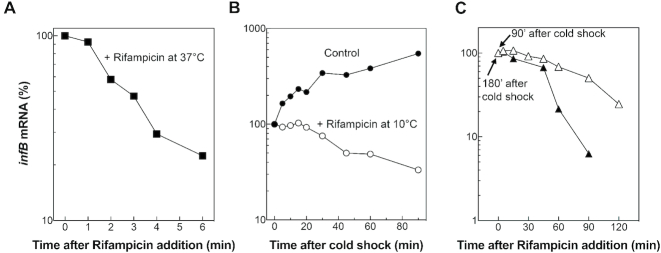
Effect of rifampicin on the *infB* mRNA levels before and after cold shock. (**A**) Variation of *infB* mRNA levels after addition of rifampicin to cells incubated at 37°C (▪). (**B**) *infB* mRNA levels in cold shocked cells treated with rifampicin (○) or not treated (•) at the onset of the cold acclimation as a function of time elapsed after rifampicin addition. (**C**) Variation of *infB* mRNA levels during cold acclimation in cells treated with rifampicin 90 min (△) and 180 min (▴) after the temperature downshift. The *infB* mRNA steady state level detected just before rifampicin addition is taken as 100%. The RNA mean half-lives (*t*_1/2_ ± standard deviation) determined from this and from two similar experiments are given in the text.

A small amount of *infB* transcript is clearly visible in the cells harvested during the exponential phase of growth in LB at 37°C (Figure 2B inset) and is sufficient to determine its chemical half-life; this was estimated to be *t*_1/2_ = 2.13 ± 0.27 min (Figure 4A), a value that can be regarded as more or less typical for bacterial mRNAs (32). Overall, these data suggest that the low level of *infB* mRNA at 37°C cannot be attributed to a particular instability of the transcript but is likely due to a low activity of the promoter(s) that control its synthesis (Figure 1).

To investigate whether the elevated level of *infB* mRNA after cold stress could be due to an increased stability, its half-life was measured during cold acclimation in three independent experiments. A strong stabilization of the *infB* mRNA was indeed observed at various times during cold adaptation (Figure 4). In fact, immediately after cold stress the RNA *t*_1/2_ increases from 2.13 ± 0.27 min to ≅ 43.5 ± 4.35 min and increases further during cold adaptation reaching its maximum value (*t*_1/2_ ≅ 97 ± 7 min) 90–120 min after the stress. Subsequently, 180–240 min after the temperature downshift, when the acclimation phase is nearing its end, the RNA level displays a biphasic decrease. In fact, whereas in the first period (from 180 to about 220 min) the RNA stability remains unchanged, after 220 min and during the following hour of cold acclimation, the*t*_1/2_ decreases to reach a level similar to that measured immediately after the stress. This biphasic behaviuor (Figure 4C) likely reflects the modification of the degradosome known to occur at the end of cold acclimation (46–49) and which should take place between 3.5 and 4 h after the cold stress in our case.

That cold stress also induces *de novo* transcription of *infB* can be clearly deduced from the fact that the immediate and steep rise of the *infB* mRNA level occurring after cold stress is not observed in cells treated with rifampicin (Figure 4B). Thus, taken together the above results indicate that the high increase of the *infB* mRNA level in cold-shocked cells results from *de novo* transcription as well as from a substantial increase of the RNA stability.

### Transcriptional activity of *nusA-infB* promoters

**Figure 5.**
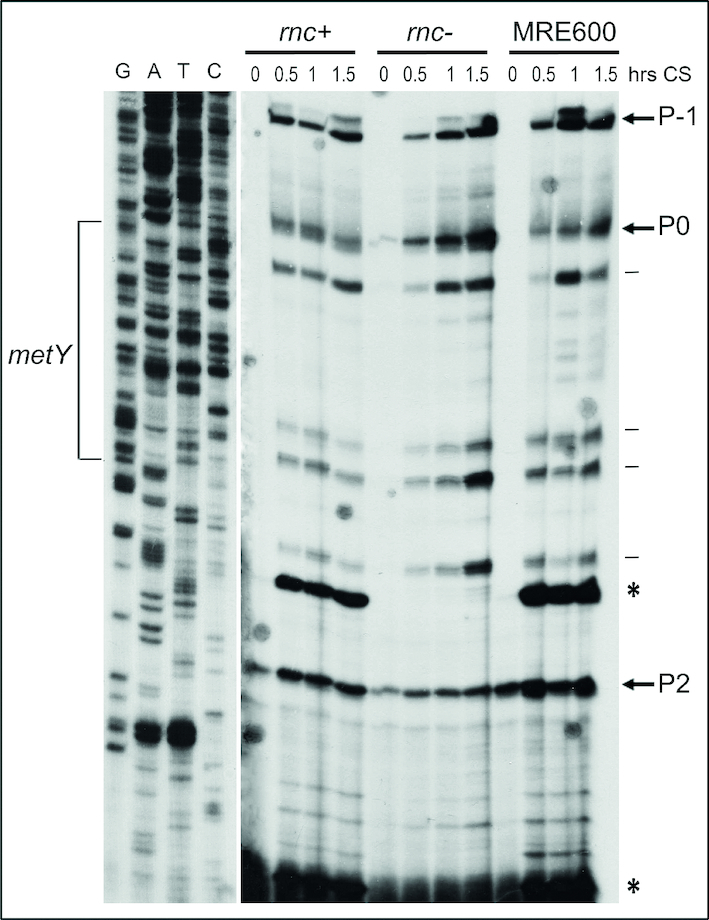
Primer extension analysis of the *nusA-infB* transcripts. The analyses were performed on total RNA extracted before and after the indicated times following a 37 to 10°C temperature downshift from cells expressing (*Escherichia coli* K12 MG1673 and *E. coli* MRE600) or not expressing (*E. coli* SK7622) the *rnc* gene that encodes RNase III as specified. The extensions originate from primer ‘a’ (Figure 1) complementary to a region downstream of RNaseIII cleavage sites. The arrows indicate the start sites corresponding to the 5’ ends of mRNAs originating from promoters P-1, P0 and P2. The two strong signals, corresponding to mRNA molecules resulting from RNaseIII processing are indicated by asterisks. Spurious stops are indicated by horizontal lines. The start sites (bold letter) and the sequences of core region of the three promoters are: P-1:ACGTTGACAAAATGTGGCATGGATCACTATAATGCCTGC**A**GATT; P0: TATTTGCATCTTTTACCCAAAACGAGTAGAATTTGCCACGTT; P2: ACTTTCCCTTAGAGTCCTTTTTCAAATATACTGTGAAGACTT

At least three promoters (P-1, P0 and P2) have been identified and characterized in the *nusA-infB* operon (15,20,30) (Figure 1). The observed cold-stress induction of *infB* transcription (Figure 2B; Supplementary Figure S2 and S3) prompted us to investigate the nature of the cold-sensitive promoters responsible for the increased expression of *infB*. To determine which of the above-mentioned promoter is active before and after cold stress, total RNA extracted from *E. coli* MRE600, *E. coli* K12 MG1673 (both RNase III^+^) and *E. coli* SK7622 (RNase III^−^) was subjected to primer extension analyses to identify the start point/s of the RNAs transcribed from the *nusA-infB* operon. The region adjacent to the *metY* gene (i.e. between *argG* and *rimP*), which contains the three main promoters of the operon, was scrutinized using primer ‘a’ (listed in ‘Materials and methods’ section), which is complementary to the DNA sequence whose location is indicated in Figure 1. The electrophoretic pattern thus obtained (Figure 5) is quite complex as it shows specific arrests corresponding to the location of the three promoters along with some signals likely due to the presence of RNaseIII-processed molecules that yield tRNA_i_ (20), and to spurious stops occurring when the AMV-RT enzyme synthesizes extremely long cDNA molecules. In any event, it is clear from the results that all three promoters are active in directing transcription in both control and in cold-shocked cells and that their activity is substantially increased during cold acclimation. It should be noted that in the pre-cold shock controls (lanes 0) primer extension arrests corresponding to transcripts originating from P0 and P-1 are very weak and could be detected only upon overexposure of the gel. The weakness of these signals can be attributed, at least in part, to the very rapid excision of the *metY* transcript that results in the rapid disappearance of the proximal regions of the primary transcripts (20). However, the analyses carried out with transcriptional fusions (see below) clearly show that both P0 and P-1 are transcriptionally active in the pre-cold shock control cells.

To evaluate the contribution of P-1 and P0 to cold-shock-induced transcription without interference from RNaseIII cleavage that yields the tRNA_i_ (20), primer extension analysis was also carried out using total RNA extracted from cells that do not express RNaseIII. The results of this experiment confirm that the transcripts originating from P-1 and P0 are scarcely present in the extracts of the cells growing at 37°C but are strongly increased after the cold stress. Comparison of the results of primer extension analyses performed on RNA extracted from *E. coli* strains expressing or not RNaseIII (i.e. *rnc*^+^ and *rnc*^−^) both before and after cold stress shows that in the absence of RNaseIII activity the intensity of the RT arrest band corresponding to P0 increases much more than those of P-1 and P2, indicating that the RNaseIII-processed molecules are mainly those transcribed from P0 (Figure 5).

Primer extension analyses like those presented above can identify the origin of transcription but do not represent a suitable and reliable method to quantify the amount of the individual transcripts. For this reason, the limited amount of transcripts originating from P-1 and P0 at 37°C and the large increase observed after cold shock should not be taken as an indication that these two promoters are hardly active in control cells and that they display a strong response to the stress. In fact, the transcripts from P-1 and P0 would begin with *metY* and our data, as well as published data (20), indicated that the product of *metY* is rapidly excised by RNaseIII to yield tRNA_i_ so as to reduce substantially the number of RNA molecules beginning at these two promoters, whereas in the absence of RNaseIII activity other RNases (e.g. RNaseE) could take over the RNA processing procedure (30). On the other hand, RNA maturation by RNaseIII (at least the maturation of rRNA precursors) is drastically slowed down after cold stress (49), a situation which would produce a large increase of the number of RNA molecules beginning at P-1 and P0 thereby leading to the incorrect consideration that these promoters are very sensitive to cold-shock induction.

### Strength and cold-responsiveness of the isolated *nusA-infB* promoters

**Figure 6.**
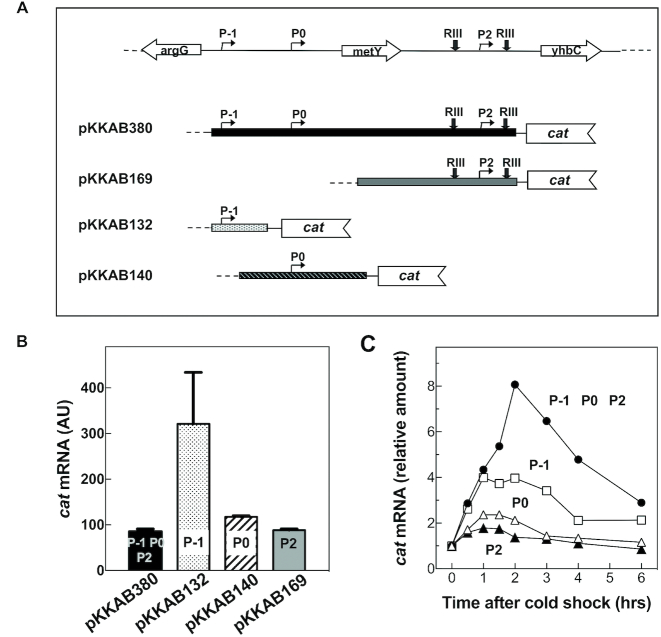
Expression of *cat* reporter gene fused to different DNA fragments derived from the *nusA-infB* operon. (**A**) Representation of the *cat* transcriptional fusion constructs used in this study. The DNA fragments schematically indicated in the figure, each containing chromosomal regions corresponding to the different promoters, were cloned upstream of promoter-less *cat* gene in pKK232–8; (**B**) Levels of *cat* mRNA expressed at 37°C in cells harbouring plasmids carrying the transcriptional fusions shown in panel (A) and containing the indicated promoters; (**C**) Relative increase of the steady-state levels of *cat* mRNA present in cells carrying the constructs containing all three promoters (P-1, P0 and P2) (•) or only P-1 (□), P0 (△) and P2 (▴) at the times after cold shock reported in the abscissa. The RNA level at time zero is taken as = 1.

In light of the above considerations, to assess the strength of the various promoters and their cold-stress responsiveness, the activities of P-1, P2 and P0 promoters were analysed outside the context of the *nusA-infB* operon. For this purpose, transcriptional fusions to the *cat* reporter gene were generated by inserting DNA fragments containing different combinations of the three *nusA-infB* promoters into the polylinker upstream of the promoter-less *cat* gene of pKK232–8 (Figure 6A). To be reliable, the quantitation of promoter strengths based on the amount of *cat* transcript expressed by the transformed cells as determined by northern blot analyses (Figure 6B and C) requires preliminary evidence that the transcripts originate from the promoters of the operon and not from other promoter-like plasmid sequences present in the constructs that might also respond to temperature changes. Therefore, primer extension analyses were carried out on RNA extracted from cells harbouring the various constructs. These experiments showed that the *cat* transcripts originated exclusively from the known operon promoters and gave no evidence for the occurrence of spurious origins of transcription either before or after cold stress (Supplementary Figure S1). In light of these results, the quantitation of the northern blots can be taken as being a reliable measure of the strength of the individual *nusA-infB* promoters. Preliminary experiments demonstrated that all transformed cells were able to grow in the presence of up to 30 μg/ml CAM. Subsequently, northern blot analyses on total RNA extracted from control and cold-stressed cells indicated that at 37°C the amount of *cat* transcript present in cells transformed with pKKAB380, which contains all three promoters, is rather low (Figure 6B) and only slightly higher in cells transformed with pKKAB140 and pKKAB169 that contain P0 and P2, respectively (Figure 6B). On the contrary, a fairly high level of *cat* transcript was detected in cells transformed with pKKAB132 (Figure 6B) that contains only P-1 that was therefore confirmed to be a very strong promoter (31).

Analysis of the cold responsiveness indicated that P-1 is also the most cold-stress responsive promoter, the *cat* transcript being increased almost 4-fold above its already high basal level in cells transformed with pKKAB132. By contrast, transcription from P0 and P2 appears to be not only quantitatively marginal at 37°C, but also much less responsive to cold stress. However, the largest increase (∼8-fold) in the *cat* transcript level was detected in cold-stressed cells harbouring pKKAB380 that contains all three promoters (Figure 6C). This increase is larger than that observed with the individual promoters so that it can be concluded that the effects of transcription induction of the three promoters are additive. Overall, it seems clear from these results that whereas P-1 is the promoter that displays the highest degree of cold-induction, also P0 and P2 are activated by the stress so that all three promoters play a synergistic role in increasing the level of *cat* transcription.

### Identification of two new cold-responsive promoters in the *nusA-infB* operon

As seen above (Figure 3A), northern blot analysis carried out on RNA extracted from control and cold shocked cells revealed the presence of two unexpected RNA molecules: one, whose size approximately corresponded to a transcript encoding NusA and IF2, and another having the size expected for an mRNA encoding only IF2. The detection of these RNAs suggested the existence of two promoters that had never been detected before located upstream of *nusA* and upstream of *infB*, respectively.

**Figure 7.**
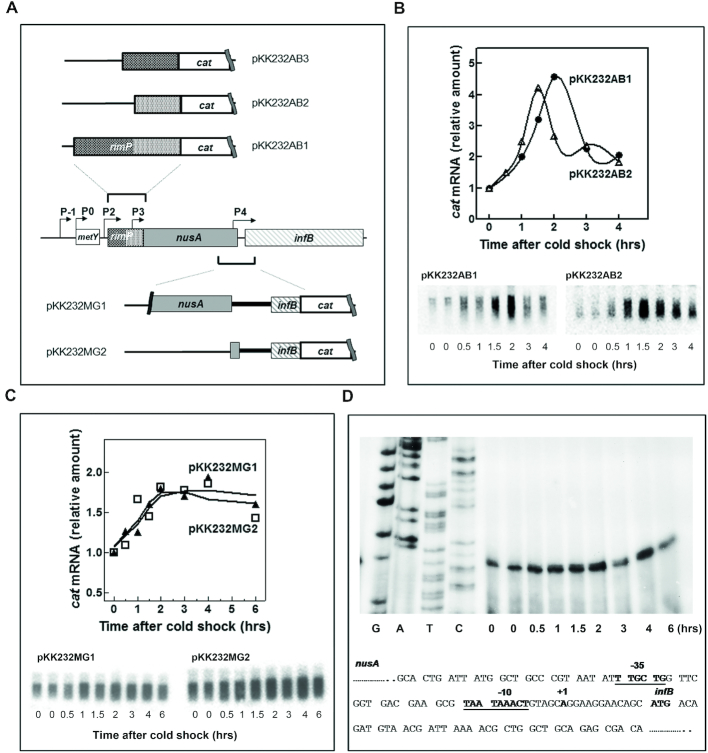
Identification of P3 and P4 two new cold-inducible promoters in the *nusA-infB* operon. (**A**) Representation of the *cat* transcriptional fusion constructs used for the identification of new promoters. The chromosomal DNA fragments used in the transcriptional fusions with the *cat* gene are indicated in the panel. The fragments corresponding to the entire *rimP* and to the proximal and distal regions of the same gene and those corresponding to the *nusA-infB* intragenic regions are schematically indicated above and below the *nusA*-*infB* operon, respectively. (**B**) Steady state levels of *cat* mRNA present before and after the indicated times of cold stress in cells transformed with pKK232AB1(•) and pKK232AB2 (△). The RNA levels were estimated from the quantification of the hybridization bands detected by northern blotting like those shown below the graph. (**C**) Cold stress-dependent increase of the steady state levels of *cat* mRNA in cells carrying plasmids in which the promoter-less *cat* gene is placed downstream the proximal portion of *infB* plus a long (pKK232MG1, ▴) or a short (pKK232MG2, □) DNA segment belonging to the distal region of *nusA*. (**D**) Primer extension analysis of the start point of the transcript originating from P4, the promoter located in the *nusA-infB* intragenic region whose sequence is reported below. The identified core elements of the P4 promoter and the first transcribed base are indicated in bold letters. The curves shown in panels (B) and (C) have been fitted using the fitspline/lowess program (Graphpad Prism).

The possible existence of the first promoter was sought by ligating a DNA fragment corresponding to the entire *rimP* gene (pKK232AB1) in front of the promoter-less *cat* gene in pKK232–8 (Figure 7A). This construct was tested for its capacity to allow the growth of the corresponding *E. coli* DH5 transformants in the presence of CAM. As seen in Supplementary Figure S5, these cells grew well in the presence of CAM and northern blot analysis showed that they contain the *cat* transcript whose level substantially increases during cold adaptation (Figure 7B). This finding indicates that a promoter is present within the *rimP* sequence. To localize better this promoter, the cells were transformed with two plasmids and bearing the distal (pKK232AB2) and the proximal (pKK232AB3) portions of *rimP*, respectively (Figure 7A). Only the cells transformed with the former construct grew (Supplementary FigureS5) and expressed the *cat* transcript; the level of this transcript was found to increase in response to cold stress (Figure 7B). These findings indicate that this newly identified promoter is located within the distal part of *rimP*. Further primer extension analyses identified a start point (Supplementary Figure S6) located 75 bp upstream of the A of the *NusA* initiation triplet and identified the likely core promoter sequences. The Pribnow box (AAAGAT) and the -35 box (ATCACA) are 3/6 and 4/6 identical to the corresponding consensus sequences (50) (Supplementary Figure S7). In light of its location downstream of the P2 promoter, we designate this new promoter P3.

Experiments similar to those described above were carried out to search for the second promoter transcribing only *infB*. In this case, two DNA fragments derived from the *nusA-infB* intracistronic region were ligated upstream of the promoter-less *cat* gene. The larger fragment (390 bp) yielding pKK232MG1 comprises the distal region of *nusA* (from -357 to +33, taking as +1 the first base of the *infB* initiation codon), the *nusA/infB* intragenic region as well as the proximal region of *infB* corresponding to the first 11 IF2 codons (Figure 7A). The shorter fragment (130 bp), which yields pKK232MG2, is similar to the other, but spans from -108 to +33 so that it contains only the most distal portion of *nusA* in addition to the *nusA/infB* intragenic region and the first 11 IF2 codons (Figure 7A). *Escherichia coli* DH5 cells transformed with either of these plasmids were able to grow at 37°C and after cold stress in the presence of CAM. Total RNA extracted from these cells before and at various times after the stress were analysed by northern blotting. A semi-quantitative estimate of the amount of *cat* transcript present in these cells indicated that between 2 and 4 h after the stress, the extent of *cat* RNA increases (∼2-fold) compared to the pre-stress level in cells harbouring either one of the two constructs (Figure 7C). Primer extension analysis performed on total RNA extracted from the cells harbouring pKK232MG1 revealed a clear arrest of the extension at a position corresponding to the A located in the *nusA-infB* intragenic region, 13 bases upstream from the first base of the IF2 start codon. In turn, the identification of this transcriptional start site allowed the identification of the core elements of the promoter (Figure 7D). Both -10 (TAATAAA) and -35 (TTGCTG) sequences identified display a fairly good correspondence with consensus sequences. Indeed, the first three bases of the -35 box and 4/6 bases of the Pribnow box correspond to the consensus and in all cases the matching bases are those that are most stringently conserved (50). We designate this promoter as P4 in light of its location downstream of the P3 promoter.

### *infB* mRNA translation

**Figure 8.**
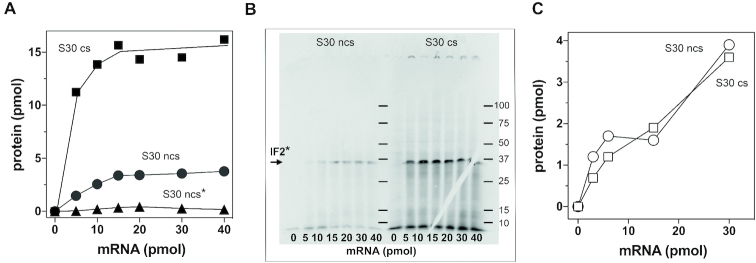
*infB* mRNA translation by extracts of control and cold-shocked cells. (**A**) Translation directed by *infB* mRNA in the amounts indicated in the abscissa by S30 extracts from *Escherichia coli* MRE600 cells subjected (▪) or not subjected (•, ▴) to cold stress. Translation was carried out for 30 min at 37°C (▪, •) or 15°C (▴). (**B**) Autoradiography of an SDS-PAGE showing the translational products obtained with the indicated increasing amounts of *infB* mRNA and S30 extracts of control cells (left) and cells subjected to cold stress (right). Further details can be found in ‘Materials and methods’ section. (**C**) Translation of bacterial phage MS2 coat protein at 37°C as a function of increasing concentrations of MS2 mRNA in the presence of cell-free extracts obtained from cells subjected (○) or not subjected (□) to cold stress. The translation conditions are the same as those presented in panel (A). The amount of protein synthesized was determined from the amount of [^35^S] methionine incorporated into hot TCA precipitated material. The experiment of panel (A) was carried out using as radioactive precursor [^35^S] methionine. Two similar experiments carried out in parallel using [^14^C] tyrosine and [^14^C] phenylalanine as precursors yielded identical results (Supplementary Figure S8).

In addition to cold-shock-induced transcriptional activation and post-transcriptional stabilization of the transcripts, it has been shown that also the translational bias, which ensures the preferential translation of the cold-shock transcripts at low temperature (see ‘Introduction’ section), contributes to expression of at least some cold shock proteins (e.g. CspA and IF1) during cold acclimation (8,9). To check if this is true also in the case of IF2, translation of *infB* mRNA was tested in cell extracts obtained from both control and cold shocked cells (Figure 8A). An autoradiography of the *infB* mRNA translation product obtained in these *in vitro* translation experiments revealed the presence of a single polypeptide (Figure 8B and Supplementary Figure S8) that is recognized by polyclonal anti-IF2 antibodies (not shown).

The translational efficiency of *infB* mRNA at 37°C was found to be substantially higher in the presence of the extract of cold shocked cells than with the extracts of control (non-cold-shocked) cells (Figure 8A), whereas comparison of the activities was not possible at low temperature because under these conditions, unlike the case of the extract of cold shocked cells that proved to be as active at 10°C as at 37°C, no translation was observed with the extracts of control cells (Figure 8A). Essentially, identical results were obtained in two parallel translation experiments in which [^14^C] tyrosine or [^14^C] phenylalanine were used as precursors (Supplementary Figure S8).

Furthermore, the experiment shown in Figure 8C shows that, unlike *infB* mRNA, a non-cold-shock mRNA such as MS2 RNA is not preferentially translated by the translational apparatus of cold-shocked cells. Thus, it is possible to conclude that *infB* mRNA is selectively subject to positive translational bias by the translational apparatus of cold-shocked cells, just like other cold-shock gene transcripts.

In addition to the *infB* mRNA subjected to positive translational bias (Figure 8A) that roughly corresponds to the transcript originating from P4, other IF2 encoding transcripts are present in the cell. However, we have not investigated if also these mRNAs are preferentially translated by the translational apparatus of cold shocked cells and if the extent of their response is similar to that observed with the mono-cistronic *infB* mRNA.

Overall, the above findings provide evidence that the cold shock translational bias not only contributes to the expression of IF2 after cold stress, but is actually necessary for *infB* mRNA translation during cold acclimation. Finally, it should be mentioned that the size of the translation product of *infB* mRNA (∼34 Kd) is substantially smaller than that of full-length IF2. There are several possible explanations for this result. A likely possibility is that the IF2 molecule undergoes cleavage upon synthesis. In fact, there is a protease-sensitive site at the border between the N terminal domain and the G domain of IF2 (51), and the size of the proteolytic product (the 294 residues-long N-terminal domain) corresponds to the size of the product synthesized *in vitro*. Another possibility is that unfavourable codons or structure of the mRNA slow down the progress of translating ribosomes and cause a premature translational arrest. On the other hand, the possibility that the mRNA is cleaved during the incubation seems to be rather remote because most of the *infB* mRNA used to program the cell-free *in vitro* translation were found intact at end of the incubation.

## DISCUSSION

Under normal conditions, growth rate control ensures the coordinated expression of ribosomal proteins whose expression in turn is geared to that of the initiation factors IF1, IF2 and IF3 whose levels are set to be approximately 7-fold lower compared to that of ribosomal proteins (13,52). However, the coordinated production of ribosomes and initiation factors is lost after cold stress, when the level of the factors is substantially increased with respect to ribosomes whose synthesis and assembly are drastically slowed down (53). On the other hand, mutual coordinate expression of the three factors is maintained even after cold stress so as to keep their stoichiometry approximately equimolar. The mechanism responsible for maintaining this coordination is not obvious insofar as the genes encoding the factors have different organizations and are located at distant positions on the *E. coli* chromosome. *InfA* (at 20 min) is a monocistronic gene controlled by two promoters (P1 and P2) (54), whereas both *infB* (at 69 min) and *infC* (at 38 min) belong to complex polycistronic operons. In *E. coli*,*infC* is located in a transcriptional unit that contains two other genes (*rpmI* and *rplT*) encoding ribosomal proteins L35 and L20, respectively (55) and is transcribed from three promoters: P_0_ and P_0′_ both located within *thrS*, the gene encoding threonyl-tRNA synthetase and P_thrS_, located upstream *thrS* (56), which allows the co-transcription of *thrS* and *infC* (57). Finally, as discussed here, *infB* is present within a cluster of genes encoding proteins involved in transcription and translation that are organized in two operons with counterclockwise orientation, the *metY-nusA-infB* and the downstream *rpoS-pnp* operon.

By means of a number of experimental approaches such as semi-quantitative determination of protein levels by western blotting, primer extension analyses and northern blotting of the transcripts using probes complementary to highly extended regions of the genes under scrutiny, here we have demonstrated that both IF2α and IF2β levels increase after cold stress, concomitantly with an increase of the steady state levels of the transcripts of the entire *metY-nusA-infB* operon including RimP and the initiator tRNA (tRNA_2_^fmet^) that had never been reported as being cold-shock inducible. These increases can be attributed to an increased activity of all the operon-proximal promoters (Figure 1). However, in this connection, it should be mentioned that previous studies (58–60) in which the transcriptomes of *E. coli* were analysed before and after cold shock had failed to detect an increased level of *infB* mRNAs as well as of other mRNAs transcribed from the *nusA-infB* operon and also from other known cold-shock genes such as *pnp* (61), *hns* (62), *infA* (9) and *infC* (10). These results, inconsistent with the detected increase of the levels of the corresponding proteins, were explained with some intrinsic shortcomings of the high density array systems used (58).

Because the sequences of the P-1 core elements match completely the consensus sequences (50), the activity of this promoter was expected to be higher than that of P0 and P2. However, this prediction proved valid only when the transcriptional activity was measured in transcriptional fusions containing the isolated promoter whereas in the presence of the other two promoters, the P-1 activity was strongly reduced. This finding suggests the existence of a strong competition between these promoters for RNA polymerase binding. Probably, this is primarily due to a competition between P-1 and P0 for RNA polymerase binding since their close proximity would prevent the simultaneous binding of two polymerases. On the other hand, P-1 proved to be the most cold-responsive promoter, and its activity being increased about 4-fold; however, the highest transcriptional activity was observed when all three promoters were present in the transcriptional fusion and the extent of the observed cold-induction indicated that the activities of P-1, P0 and P2 become additive under the experimental conditions.

To the best of our knowledge, not only the stable RNAs (rRNAs and tRNAs), but also the genes encoding the components of the translational apparatus are subject to growth rate control and to inhibition by guanosine 5′ diphosphate-3′diphosphate (ppGpp). Thus, although not directly demonstrated, it is logical to assume that also *infB* transcription is subject to the same regulatory mechanisms as the other components of the translational machinery. The presence of a stringent box within the P-1 promoter is consistent with this assumption. Cold stress is accompanied by a reduction of the cellular concentration of pppGpp and ppGpp, and the expression of cold-shock proteins is diminished and increased by high and low levels of ppGpp, respectively (63). It seems therefore likely that the promoters that display the highest cold-stress induction are those that are more sensitive to ppGpp inhibition during growth at 37°C.

In light of this consideration, it is possible to explain the high level of cold-stress induction displayed by P-1. In fact, unlike all the other promoters of the operon, the sequence of P-1 downstream the -10 element and before the +1 Adenine has the typical character of a promoter subject to stringent control with 5 out of 6 bases being GCs (underlined in Supplementary Figure S9) and therefore subject to inhibition by ppGpp (64,65).

The difference in cold-induced expression of *metY* and *metZ* in the K12 and MRE600 strains (Figure 3B) deserves some consideration. Unlike that of *E. coli* K12, the sequence of this region of the chromosome was not known for *E. coli* MRE600. Thus, in this study the relevant sequence for this B strain was obtained and compared to that of the K12 strain (Supplementary Figure S9). The two sequences are almost identical, but for one base in the spacer region of P-1 and one in the spacer region of P0. While the difference in the latter case is likely irrelevant insofar as it concerns the base immediately downstream the -35 element whose nature seems to be random, in the case of P-1 the difference is in the middle of the spacer, in a region crucial for determining the spacer structure. Indeed, it has been shown that differences of the sequence in this part of the spacer may affect the structural properties of DNA such as flexibility and curvature and, with that, the transcriptional efficiency of the promoter (66–68). It is therefore tempting to attribute to this difference, the different behaviour of the initiator tRNA genes when the two *E. coli* strains are cold shocked.

Because northern blot analysis of the transcripts of the *nusA-infB* operon and hybridization with specific probes revealed the presence of RNA molecules that could originate from so far unidentified promoters, experiments were specifically designed to detect their possible existence. Thus, promoter activity observed in select segments of the operon allowed us to identify two new cold-responsive promoters that we designate P3 and P4. Promoter P3 is located in the distal portion of *rimP* and its activity would be responsible for the transcription of an RNA encoding *nusA* and terminating somewhere within the *infB* gene. Promoter P4 was localized within the intragenic region between *nusA* and *infB* and would be responsible for the expression of *infB* as a monocistronic mRNA. The level of the transcripts originating from P3 and P4 appears low at 37°C but increases substantially during cold acclimation indicating that both promoters are cold-inducible. The -10 and -35 core elements of both newly identified promoters display a good correspondence with the established consensus sequences (50).

It has been proposed that the cold-shock induction of *nusA, infB, rbfA* and *pnp* occurs through transcription anti-termination mediated by CspA and other cold shock-induced Csp proteins (69). However, our data are not consistent with this premise. In fact, anti-termination should result in the appearance of new species of RNA, something which our northern blot analyses do not reveal since the patterns of the transcriptional products before and after cold stress are very similar without any indication of relevant transcriptional read-through events.

Particularly relevant for the increased synthesis of IF2 after cold stress is the induction of P4 that generates an mRNA molecule specifically devoted to the expression of this protein. However, the cold stress increased expression of IF2 does not arise exclusively from an increased transcriptional activity but also from two types of post-transcriptional regulations. Indeed, an ∼35-fold increase of the *t*_1/2_ contributes to increase the *infB* mRNA level during cold acclimation. This stabilization is substantially larger than what results from plain temperature decrease and larger than that observed with *infA* mRNA (∼10-fold) (9) albeit much less dramatic than reported for the *cspA* transcript whose *t*_1/2_ increases >100-fold (70). As with the *cspA* mRNA, also the *infB* mRNA stabilization is transient being reversed when the cells become cold adapted, concomitantly with the change in the composition of the degradosome that occurs at the end of cold acclimation (46–48). Finally, translation of the *infB* mRNA by the cold-stress translational apparatus seems to be a *conditio sine qua non* for the successful synthesis of IF2 during cold acclimation. Concerning this point, our data demonstrate that like *hns, cspA, infA* and *hupB* mRNAs (6–10,71), *infB* mRNA is just another cold-shock mRNA subject to a positive translational bias by the translational apparatus of cold shocked cells.

**Figure 9.**
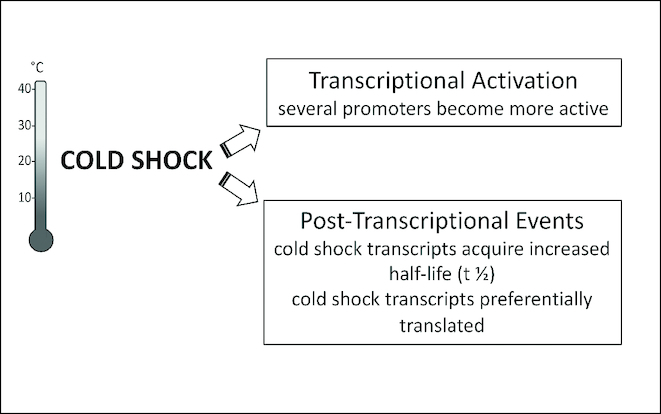
Scheme summarizing the transcriptional and post-transcriptional events triggering *de novo infB* expression in cold stressed *Escherichia coli* cells.

The present data highlight both similarities and differences between the mechanisms that ensure cold-stress induction of IF2, IF1 and IF3. Increased transcriptional activity, stabilization of the transcripts and preferential translation of the corresponding mRNAs are common features but, aside from these general similarities, the relative importance and some details of these three mechanisms appear different in the three cases. With IF1 mRNA stabilization seems to play a marginal role whereas a major role appears to be the rather selective transcriptional activation of an otherwise less active promoter that generates a longer transcript whose translation is preferentially favoured by the translational bias. For IF3, aside from transcriptional activation of the *infC* gene, the loss of the translational auto-repression at low temperature seems to play a major role. Finally, the increased level of IF2 seems to rely equally on all three mechanisms, as summarized in Figure 9.

## Supplementary Material

Supplementary Data

## Notes

Present address: Anna Brandi, School of Biosciences and Veterinary Medicine, University of Camerino, 62032 Camerino (MC), Italy.

Present address: Mara Giangrossi, School of Biosciences and Veterinary Medicine, University of Camerino, 62032 Camerino (MC), Italy.

Present address: Roberto Spurio, School of Biosciences and Veterinary Medicine, University of Camerino, 62032 Camerino (MC), Italy.

Present address: Anna M. Giuliodori, School of Biosciences and Veterinary Medicine, University of Camerino, 62032 Camerino (MC), Italy.
