# Supplementary material for: Transcriptional and post-transcriptional events trigger de novo infB expression in cold stressed Escherichia coli
Source: Nucleic Acids Res. 2019 Mar 27;47(9):4638–51. doi: 10.1093/nar/gkz187 (PMC6511841; doi:10.1093/nar/gkz187)
Supplement: Supplementary Data [file gkz187_supplemental_files.pdf]

Supplementary Data

TableS1: Sequence of oligonucleotide pairs used for probes synthesis

Target	Forward primer	Forward primer	length	probe
yhbC	5'- GGTGGGCTTGTCCACATTAG -3'	5'- AGCGCGAACACTTCATCTTT -3'	421	I
nusA	5'- **T**AGTCTGGATGAGGTGAAAAGC -3'	5'- GAATAGAGCACGCCACGAAC -3'	581	II
infB	5'- ATAAACTGTAGCAGGAAGGAACAG -3'	5'- CGTTATCAGTCCATTTGTTTTCTT -3'	680	III
rbfA	5'- TTTGGATCAGGAGAATTTATTATGG -3'	5'-GCAACGTTGAGGACGACTCATTA-3'	440	IV

Table S2: Sequence of oligonucleotide pairs used in PCR reactions

Recombinant plasmid	Forward primer	Reverse primer	Promoters	Annealing Temp.
pKKAB380	5'- AAGGCAAAAGTATTCAACAAAT -3'	5'- TAACTGAACCCTATAACCGCAAC -3	P-1, P0, P2	56°C
pKKAB169	5'-GTAGCTCGTCGGGCTCATAAC-3'	5'- TAACTGAACCCTATAACCGCAAC -3'	P2	55°C
pKKAB140	5'-CCGTCTCGGTACACCAAATC -3'	5'- AACCGACGATCTTCGGG -3'	P0	54°C
pKKAB132	5'- AAGGCAAAAGTATTCAACAAAT -3'	5'-GGGATTTGGTGTACCGAGAC-3'	P-1	55°C
pKK232AB1	5'-GGTGGGCTTGTCCACATTAG-3'	5'-CGCAAGCTTTTCACCTCATCCAG-3'	All *rimP*	54°C
pKK232AB2	5'-AAGGATCCTCACCGGGTCTCG-3'	5'-CGCAAGCTTTTCACCTCATCCAG-3'	Distal *rimP*	56°C
pKK232AB3	5'-GGTGGGCTTGTCCACATTAG-3'	5'-CGCAAGCTTAGTGTTCAGCCGTG-3'	Proximal *rimP*	56°C
pKK232MG1	5'-GCGGATCCAAGAGCTGTTGGAAATCGAAG-3'	5'-TCTAAGCTTCTGCAGCCAGCGTTTTAATC-3'	*nusA-infB*	58°C

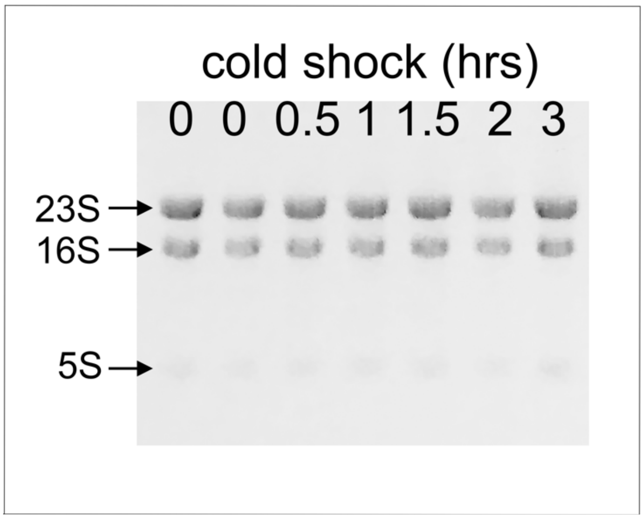

Figure S1 Agarose (1%) gel electrophoretic separation of total RNA (4 μg/lane) extracted from cells subjected to cold shock at 10°C for the indicated times and stained with ethidium bromide. The positions of 23S, 16S and 5S rRNA are indicated by the arrows.

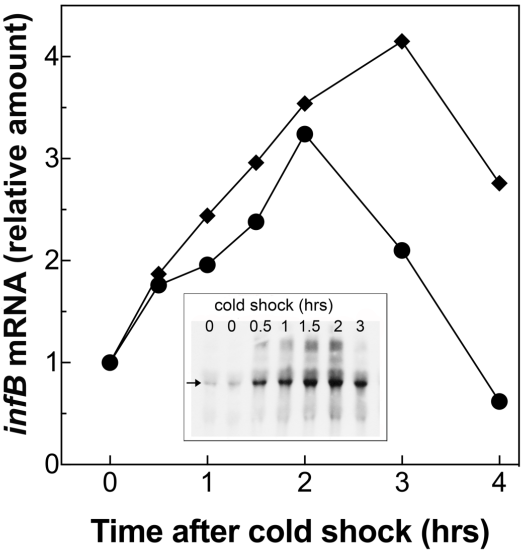

Figure S2 Northern blot analyses of *infB* mRNA present in *E. coli* MRE600 cells subjected to cold shock at 10°C for the times indicated in the abscissa. The Y-axis shows the relative increase of the RNA with respect to the pre-shock level taken as =1. The results of two separate experiments are presented (Exp.1◆; Exp. 2 ●). The inset presents a typical Northern blot from which data were quantified. Lanes marked 0 are duplicate samples taken before the stress. The arrow indicates the *infB* mRNA.

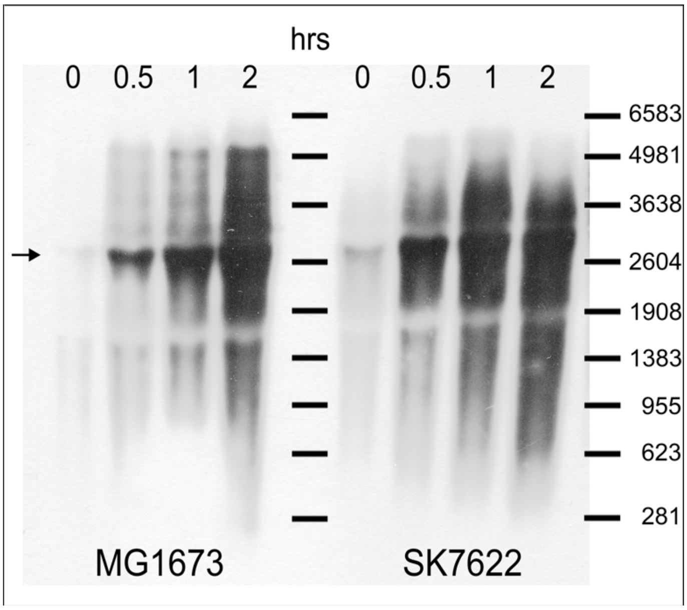

Figure S3 Northern blot analyses of *infB* mRNA present in two K-12 derivatives of *E. coli* (MG1673 left and SK7622 right) subjected to cold shock at 10°C for the times indicated above each lane; 0 represents the samples taken just before the stress.
Matching the main *infB* mRNA band (indicated by an arrow) with the RNA size ladder in the middle and on the right side of the gel yields a size of ca. 2,700 nucleotides which corresponds well to the expected size of an mRNA exclusively encoding IF2α (891 amino acids).

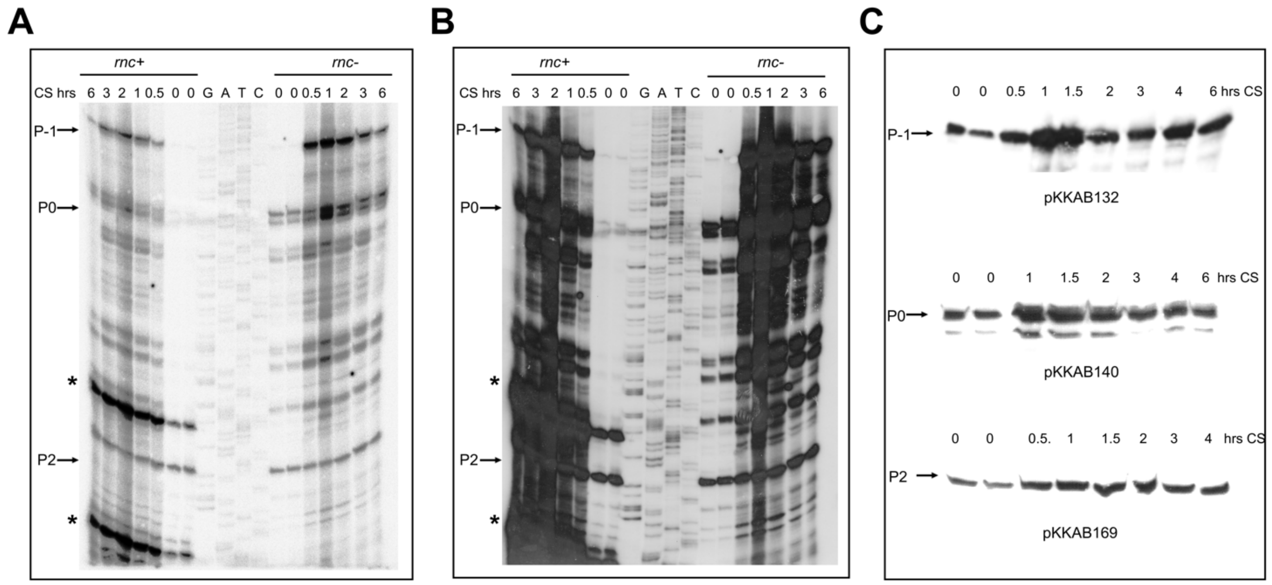

Figure S4 Determination of transcription start sites in pKK constructs**.** (A) Primer extension analysis was carried out on total RNA extracted from *E. coli* wt (*rnc*$^{+}$) and *rnc*$^{-}$ (as indicated above the gel) carrying the recombinant plasmid pKKAB380 (containing promoters P-1, P0, P2). The numbers above the individual lanes indicate the time elapsed after cold shock at 10°C;(B) this panel shows the same gel presented in panel (A) exposed for a longer period to allow a better visualization of the G, A, T, C sequencing lanes in the centre. (C) Analysis of the start sites of the RNAs synthesized from the individual P-1 (pKKAB132), P0 (pKKAB140) and P2 (pKKAB169) promoters before and after cold shock in wt cells. In all panels the start sites are indicated with arrows whereas the beginning of RNA molecules processed by RNaseIII are indicated by asterisks. The times after cold shock (hrs) are indicated above each lane. To detect only transcripts resulting from transcriptional fusions, an oligonucleotide “b” (see Materials and Methods) complementary to a region downstream of the plasmid multi-cloning site was used as primer.

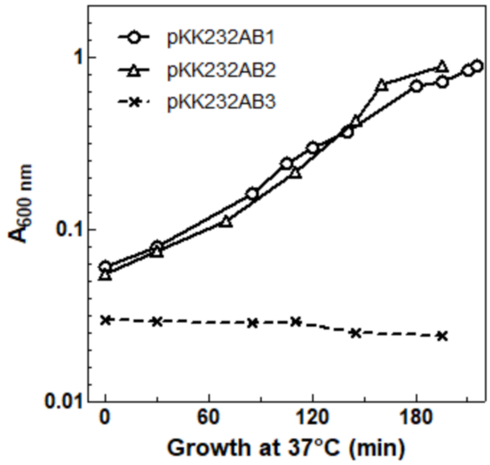

Figure S5 Growth at 37°C in LB medium containing CAM (30μg/ml) of *E. coli* DH5α cells transformed with pKK232AB1, pKK232AB2 and pKK232AB3 in which the promoter-less *cat* gene is placed downstream the entire *rimP* (○) or the distal (△) or proximal (X) part of *rimP*.

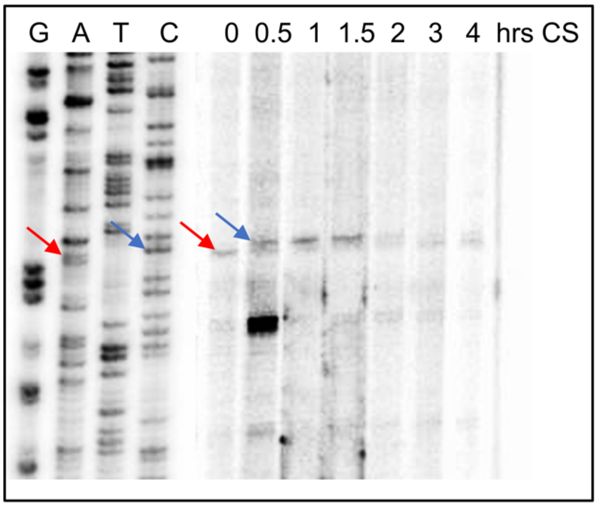

Figure S6 Primer extension analysis within the region of the P3 promoter of the transcripts present in *E. coli* DH5α carrying pKK232AB2 subjected to cold shock at 10°C for the times indicated above each lane. The two detected arrest sites are indicated with arrows. The strong band present exclusively in the 0.5 hour time point is due to a spurious arrest.

AAGGATCCTCACCGGGTCTCGATCGCCCACTGTTCACGGCTGAACACTACGCCCGTTTTGTCGGAG

AAGAGGTGACTCTGGTTCTCCGTATGGCGGTACAAAACCGTCGTAAATGGCAGGGCGTTATCAAAG

-35 -10 +1

CGGTAGACGGTGAAATG**ATCACA**GTTACCGTCGAAGGT**AAAGAT**GAAGT**GT**TCGCGCTGAGTAATA

TCCAGAAGGCGAACCTGGTTCCCCACTTT**TAA**TAGTCTGGATGAGGTGAAAAGC**ATG**AACAAAGAA

Figure S7 Sequence of the distal portion of *rimP* containing the P3 promoter. The core elements of the promoter are in bold letters and underlined. The two identified transcriptional start points are in bold letters under the same +1 indication. The TAA termination codon of *rimP* mRNA is indicated in bold letters. The sequence of *nusA* mRNA is written in grey and its start codon is boxed.

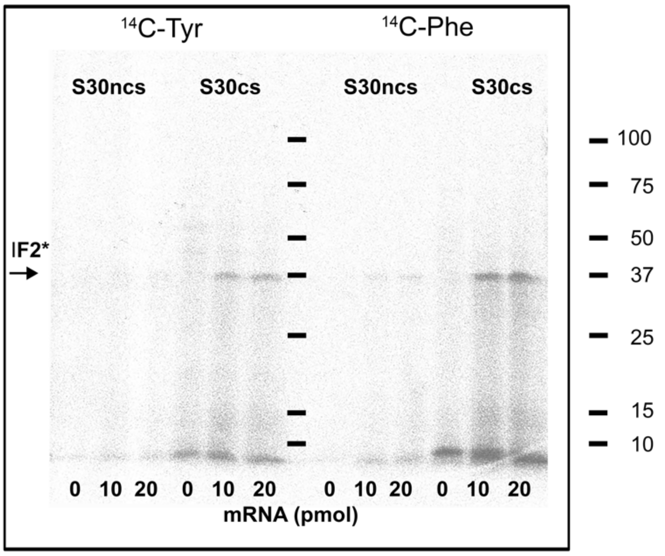

Figure S8 Electrophoretic analysis of the translational products synthesized *in vitro* under the direction of the indicated amounts of *infB* mRNA in cell-free systems containing extracts of non-cold-shocked (S30ncs) and cold-shocked (S30cs) cells, as indicated. Translation was carried out in the presence of [^{14}C] tyrosine (left gel) or [^{14}C] phenylalanine (right gel). A molecular weight ladder is shown between the two gels and to the right side. The arrow indicates the position of the product synthesized which is designated as IF2* to indicate that its size is smaller than intact IF2α.

```
                                                     -35
MRE600-CAAATGAAAGTGAACTGGATATTCATTCACGTGATTAGCAATAAACGTTGACAAAAT
K12   -CAAATGAAAGTGAACTGGATATTCATTCACATGATTAGCAATAAACGTTGACAAAAT

   P-1           -10       →
MRE600-GTGGCATGGATCACTATAATGCCTGCAGATTTTACGTCCCGTCTCGGTACACCAAAT
K12   -GTGGCGTGGATCACTATAATGCCTGCAGATTTTACGTCCCGTCTCGGTACACCAAAT

            -35       P0        -10       →
MRE600-CCCAGCAGTATTTGCATCTTTTACCCAAAACGAGTAGAATTTGCCACGTTTCAGGCG
K12   -CCCAGCAGTATTTGCATTTTTTACCCAAAACGAGTAGAATTTGCCACGTTTCAGGCG
```

Figure S9 Comparison of *E. coli* MRE600 and *E. coli* K12 sequences in the chromosome region comprising promoters P-1 and P0. The core elements of the promoters are highlighted in yellow (P-1) and grey (P0). The transcriptional starts are indicated in bold letters and by arrows, the stringent box is underlined and the sequence differences between the two bacterial strains are indicated in bold red letters.
